# High-grade glioma associated immunosuppression does not prevent immune responses induced by therapeutic vaccines in combination with T_reg_ depletion

**DOI:** 10.1007/s00262-018-2214-0

**Published:** 2018-07-27

**Authors:** Mario Löhr, Benjamin Freitag, Antje Technau, Jürgen Krauss, Camelia-Maria Monoranu, Johannes Rachor, Manfred B. Lutz, Carsten Hagemann, Almuth F. Kessler, Thomas Linsenmann, Matthias Wölfl, Ralf-Ingo Ernestus, Sabrina Engelhardt, Götz Gelbrich, Paul G. Schlegel, Matthias Eyrich

**Affiliations:** 1 Department of Neurosurgery, University Medical Center Würzburg, Würzburg, Germany; 2 Department of Pediatric Oncology, University Children’s Hospital Würzburg, Josef-Schneider-Strasse 2, 97080 Würzburg, Germany; 3 Department of Pediatric Neurosurgery, University Medical Center, Würzburg, Germany; 4 Department of Neuropathology, Institute of Pathology, University of Würzburg, Würzburg, Germany; 5 Institute for Virology and Immunobiology, University of Würzburg, Würzburg, Germany; 6 Department of Epidemiology and Biostatistics, University Medical Center, Würzburg, Germany

**Keywords:** High-grade glioma, Immunosuppression, MDSCs, Treg depletion, DC vaccines, CNS homing

## Abstract

High-grade gliomas (HGG) exert systemic immunosuppression, which is of particular importance as immunotherapeutic strategies such as therapeutic vaccines are increasingly used to treat HGGs. In a first cohort of 61 HGG patients we evaluated a panel of 30 hematological and 34 plasma biomarkers. Then, we investigated in a second cohort of 11 relapsed HGG patients receiving immunomodulation with metronomic cyclophosphamide upfront to a DC-based vaccine whether immune abnormalities persisted and whether they hampered induction of IFNγ^+^ T-cell responses. HGG patients from the first cohort showed increased numbers of leukocytes, neutrophils and MDSCs and in parallel reduced numbers of CD4^+^/CD8^+^ T-cells, plasmacytoid and conventional DC2s. MDSCs and T-cell alterations were more profound in WHO IV° glioma patients. Moreover, levels of MDSCs and epidermal growth factor were negatively associated with survival. Serum levels of IL-2, IL-4, IL-5 and IL-10 were altered in HGG patients, however, without any impact on clinical outcome. In the immunotherapy cohort, 6-month overall survival was 100%. Metronomic cyclophosphamide led to > 40% reduction of regulatory T cells (T_reg_). In parallel to T_reg_-depletion, MDSCs and DC subsets became indistinguishable from healthy controls, whereas T-lymphopenia persisted. Despite low T-cells, IFNγ-responses could be induced in 9/10 analyzed cases. Importantly, frequency of CD8^+^VLA-4^+^ T-cells with CNS-homing properties, but not of CD4^+^ VLA-4^+^ T-cells, increased during vaccination. Our study identifies several features of systemic immunosuppression in HGGs. Metronomic cyclophosphamide in combination with an active immunization alleviates the latter and the combined treatment allows induction of a high rate of anti-glioma immune responses.

**Electronic supplementary material:**

The online version of this article (10.1007/s00262-018-2214-0) contains supplementary material, which is available to authorized users.

## Introduction

More than four decades ago, Brooks and colleagues reported about reduced lymphocyte function in patients with intracranial tumors [3]. The advent of cancer immunotherapies has renewed interest in high-grade glioma (HGG)-associated immune dysfunctions, as they could limit the success of immunotherapeutic interventions.

Inhibitory features of glioma cells can result in either systemic or local immunosuppression. Whereas the latter mainly impedes the effector phase of anticancer immunity, systemic immunosuppression represents a major hurdle against a successful priming of glioma-directed immune responses. On the systemic level, an altered composition of the leukocyte compartment with an increased neutrophil/lymphocyte ratio [4] and increased frequencies of MDSCs in peripheral blood have been reported [5–7]. Several groups have described elevations of cytokines and growth factors [8–10]. Importantly, in murine glioma models it could be demonstrated that interference with immunosuppression like depletion or inhibition of regulatory T-cells (T_reg_) and MDSCs is associated with reduced gliomagenesis, increased antitumor immune responses and improved survival [11–13].

Priming of T-cell responses against glioma antigens is a prerequisite for efficacy of both therapeutic vaccinations as well as immune checkpoint inhibitors. Although spontaneous immune responses in non-vaccinated glioma patients do sporadically exist [14], the majority of patients can be considered immune naive. Clinical vaccination studies in glioma patients published so far have reported an induction of T-cell responses in 50.3% of cases (range 33–66%) [14–21], which is still far lower than vaccine response rates using viral antigens in conventional prophylactic vaccines. Depletion of T_reg_ has been demonstrated to act synergistically with immunotherapy in several murine glioma models [22, 23]. Indeed, also in human studies of other cancer entities, e.g. in renal cell carcinoma [24], similar observations have been described. In the clinical situation, metronomic cyclophosphamide has proven to be a simple, inexpensive, and well-tolerated means to deplete T_reg_ [25, 26].

In the present study, we first evaluated in a cohort of HGG patients a panel of soluble and cellular biomarkers in peripheral blood and their correlation with survival. Then we investigated in a second cohort of relapsed HGG patients, who received immunomodulation with T_reg_-depletion followed by a DC-based therapeutic vaccine, whether these alterations in cellular immunity are persisting and whether they prevent induction of anti-glioma immune responses. Our data provide a clinical rationale for the concept of using immunomodulation upfront of a therapeutic vaccine to reverse distinct immunosuppressive features in HGG which paves the way for more efficacious immune respones.

## Materials and methods

### Patients

From April 2012 to March 2014 preoperative blood samples (PBMCs and plasma) from *n* = 79 patients who underwent surgery for a suspected HGG were collected in the department of Neurosurgery at the University Hospital Würzburg, Germany. From these, *n* = 18 had to be excluded, because intra-surgical or histological findings revealed a different etiology (WHO grade I°–II° glioma, metastases, inflammatory lesions), so that *n* = 61 patients including two children and three adolescents with a confirmed WHO III°–IV° HGG remained evaluable. Details of patient characteristics as well as diagnostic and therapeutic interventions are given in Table 1 and supplementary Fig. 1. All primary disease cases were included before any tumor treatment was initiated; relapsed patients had received standard of care (surgery, radiation, temozolomide) as primary treatment, but were off-therapy ≥ 4 weeks before inclusion. Due to rapid postoperative transfer of several patients back into referring centers, a post-surgery blood sample could only be obtained in 36 cases. Blood samples from 9 healthy adults (mean age 37.2 ± 18.5 years, males/females 6/3) without any history of tumors, neurosurgery or immunmodulating medication served as a control.

**Table 1:** Patient characteristics of cohort I and II

Cohort I					
Median age (range)	60.2 years (7.4–83.8)			Median follow-up	13.7 months (8.2–36.5)

			PFS [months]	OS [months]	Alive *n* =
Total	*n* = 61		7.8	8.2	15 (25%)

			Subpopulations		
			PFS [months]	OS [months]	alive *n* =
Primary diagnosis, complete resection, radiochemotherapy	*n* = 12 (20%)		10.1	15.5	4 (33%)
< 18 years	*n* = 5 (8%)		7.9	9.2	3 (60%)
> 18 years	*n* = 56 (92%)		7.1	8.2	12 (21%)
WHO grade III°	*n* = 8 (13%)		7.1	12.1	3 (38%)
WHO grade IV°	*n* = 53 (87%)		7.8	7.8	12 (23%)
Primary diagnosis	*n* = 46 (75%)		7.8	8.2	11 (24%)
Relapsed glioma	*n* = 15 (25%)		7.4	8.4	5 (33%)
Total resection	*n* = 23 (38%)		7.8	8.6	6 (26%)
Subtotal resection	*n* = 19 (31%)		7.0	9.0	7 (37%)
Partial resection/biopsy	*n* = 19 (31%)		7.1	7.8	2 (11%)
Female	*n* = 21 (34%)		7.1	8.4	6 (29%)
Male	*n* = 40 (66%)		7.8	8.2	9 (23%)

Cohort II					
Parameter		Patients from cohort II receiving immunotherapy after 2nd surgery *n* = 11 (*n* = 6 children/adolescents, *n* = 5 adults)			
Age (mean ± SD)		26.1 ± 20.8 years			
Male to female ratio		7/4			
WHO III° vs. WHO IV°		1/10			
6-month survival		11/11 (100%)			
OS (mean ± SD)		14.9 ± 13.4 months			

A second cohort of *n* = 11 patients with relapsed HGGs (Table 1, bottom) was treated within the feasibility pilot phase of the *HIT-HGG Rez Immunovac* trial. Patients eligible for second resection received metronomic cyclophosphamide (1.5 mg/kg, max. 100 mg daily in two divided doses) from diagnosis until the day before the first vaccine. In 10 patients, a total/subtotal tumor removal was possible, and in one patient only a partial resection could be achieved. After second resection and approx. 2–3 weeks after initiation of cyclophosphamide, patients underwent an unstimulated apheresis to collect at least 2 × 10^9^ monocytes. Immunotherapy consisted of four weekly intradermal DC vaccinations in imiquimod-prepared skin, followed by three monthly boost vaccines with 1,500 µg tumor lysate (TL), and subsequent tumor lysate boosts every 3 months as long as material was available.

### Vaccine generation

DCs and TL were prepared as previously described [27]. In brief, autologous tumor material was mechanically dissected using the GentleMACS device (Miltenyi Biotec, Bergisch-Gladbach, Germany) and avitalized by six freeze–thaw cycles and 60 Gy irradiation. Monocytes were enriched from the apheresis product by elutriation and cultivated for 7 days in GM-CSF and IL-4 (1000 U/ml each). On day 7, immature DCs were counted, pulsed with tumor lysate (50 µg/10^6^ DCs) and matured for another 48 h with IL-1β (2000 U/ml) and TNFα (1000 U/ml). Culture bags, medium (CellGro^®^) and cytokines were purchased from CellGenix (Freiburg, Germany).

### Flow cytometry

Full blood counts from all samples were obtained using an automated hematology counter (Advia 2120i, Siemens, Germany). Flow cytometric assessment included the following markers: CD1c, CD3, CD4, CD8, CD11b, CD14, CD15, CD16, CD19, CD27, CD29, CD45, CD45RA, CD45RO, CD49d, CD56, CD83, CD86, CD303, HLA-DR, TCRγδ. All antibodies were obtained from BD (BD Biosciences/Pharmingen, Heidelberg, Germany) except TCRγδ (MiltenyiBiotec, Bergisch-Gladbach, Germany). Cells were stained and prepared using standard lyse/wash eight-color procedures. For identification of FoxP3^+^ T_reg_ subpopulations, the human regulatory T cell staining kit from ebioscience (Frankfurt, Germany) was used. 10,000 (for lymphocytes) or 100,000 (for DC- or MDSC-subpopulations) events were aquired on a FACSCanto II. Results were analyzed with FlowJo Software (version 9.6, TreeStar, Ashland, USA).

For analysis of MDSC-subpopulations, we concentrated on four previously described populations with a myeloid or monocytic phenotype: CD14^+^ HLA-DR^neg^ [5, 28], CD33^+^CD14^neg^HLA-DR^neg^ [29], SSC^high^CD66b^+^CD125^neg^ [30], CD66b^+^CD16^high^CD14^neg^ [31]. For the latter two populations, sideward scatter (SSC) data were collected in linear mode to allow a better discrimination of granulocytic populations [30]. A total of 30 hematologic parameters were assessed.

### Plasmacytoid dendritic cell culture

The frequency of plasmacytoid dendritic cells (pDCs) in peripheral blood was determined by flow cytometry. Vital, 7-aminoactinomycin^negative^ (7-AAD^neg^) pDCs were identified as the mean frequency of SSC^low^CD303^+^7-AAD^neg^ events from two independent tubes. PDCs with these characteristics were assayed for their expression levels of CD80, CD83, CD86, chemokine receptor (CCR) 7, chemokine ligand (CXCL) 10, and PD-L1 (CD274) by flow cytometry as described above. For functional pDC-assays, we ficollized PBMCs from 20 ml of heparinized blood and magnetically separated pDCs with blood dendritic cell antigen 4 (BDCA-4) microbeads (Miltenyi Biotec, Bergisch-Gladbach, Germany). Enriched pDCs were plated in 96-well plates supplemented with CellGro medium (CellGenix, Freiburg, Germany), IL-3 10 ng/ml and imiquimod 5 µg/ml (Sigma–Aldrich, Taufkirchen, Germany). After 48 h of culture, cells were harvested and analyzed for expression of the above mentioned markers.

### Biomarker assessment

Fresh EDTA-plasma was collected within 24 h after sampling and stored in two aliquots at − 80 °C until analysis. Plasma biomarkers were measured according to the manufacturer’s instructions on a MagPix device (Luminex, Oosterhout, The Netherlands) using the following kits: Human Magnetic 30-Plex Kit (LHC6003M, Lifetechnologies, Darmstadt, Germany) for G-/GM-CSF, IFNα/γ, IL-1β/-1RA, IL-2/-2R, IL-4, IL-5, IL-6, IL-7, IL-8, IL-10, IL12p40/70, IL-13, IL-17, TNFα, Eotaxin, Interferon gamma-inducible protein 10 (IP-10), Monocyte Chemoattractant Protein 1, Monokine Induced by Gamma interferon, MIP-1α/β, (Regulated on Activation, Normal T cell Expressed and Secreted (RANTES), Epidermal Growth Factor (EGF), Fibroblast growth factor basic, Hepatoblast growth factor, VEGF, HCCBP1MAG-58k for Osteopontin, Fas, FasL, APOMAG-62k for apolipoprotein A1, and TGFBMAG-64k-01 for TGFβ (all from Millipore Merck, Schwalbach, Germany). Luminex assays included an internal calibration set, high and low validation samples and a 7-point curve for standard generation. From the 30-Plex Kit all parameters passed the internal validation with the exception of RANTES and IL-17 (> 50% of values out of range); these two parameters were excluded from further analysis.

### Immune monitoring

T-cell responses defined by specific IFNγ-secretion were measured using a stimulation-expansion-restimulation protocol [32]: PBMCs were isolated by Ficoll, stimulated with mature, tumor-lysate loaded autologous DCs at a 4:1 (T:DC) ratio and expanded subsequently with IL-15 (5 ng/ml) for 12–14 days. Medium and IL-15 was refreshed every second or third day. On days 7 and 14 PBMCs were restimulated with the same tumor-lysate loaded DCs. Negative control was stimulation with “empty” DCs (mature, autologous DCs without tumor-lysate loading). 6 h after the last restimulation, T cells were analyzed for IFNγ-production by intracellular cytokine staining (patient 2–11). Frequencies of lysate-reactive IFNγ^+^ T-cells are given background corrected, i.e. D %IFNγ^+^ DC_TL_−%IFNγ^+^ DC_empty_.

### Statistical analysis

Biomarkers were analyzed either on the scale of measurement (assuming additive effects, i.e. mean differences) or on the logarithmic scale (assuming multiplicative effects, i.e. geometric mean ratios). The decision was made by the Shapiro–Wilk test to determine which distribution was closer to normality: measured values (supports additive effects) or logarithmic values (supports multiplicative effects). Changes from pre- to postoperative measurements were analyzed by Student’s paired *t* test. Differences between independent groups were evaluated by one-way ANOVA. Relationships between lymphocyte subpopulations and biomarkers with PFS and OS were assessed by Cox regression. Dependencies between the biomarkers were described by Spearman’s correlation coefficient. Since several clinical variables of HGGs might be associated with better outcome, we also computed the hazard ratios adjusted for a risk score counting one penalty point for WHO IV°, adult age and relapse diagnosis. *p* values < 0.05 were considered statistically significant. 95% confidence intervals (CI) were provided for effect estimates. All calculations were carried out with the statistical software SPSS, version 23 (IBM Corp).

## Results

### White blood cell subpopulations in HGG patients

**Fig. 1:**
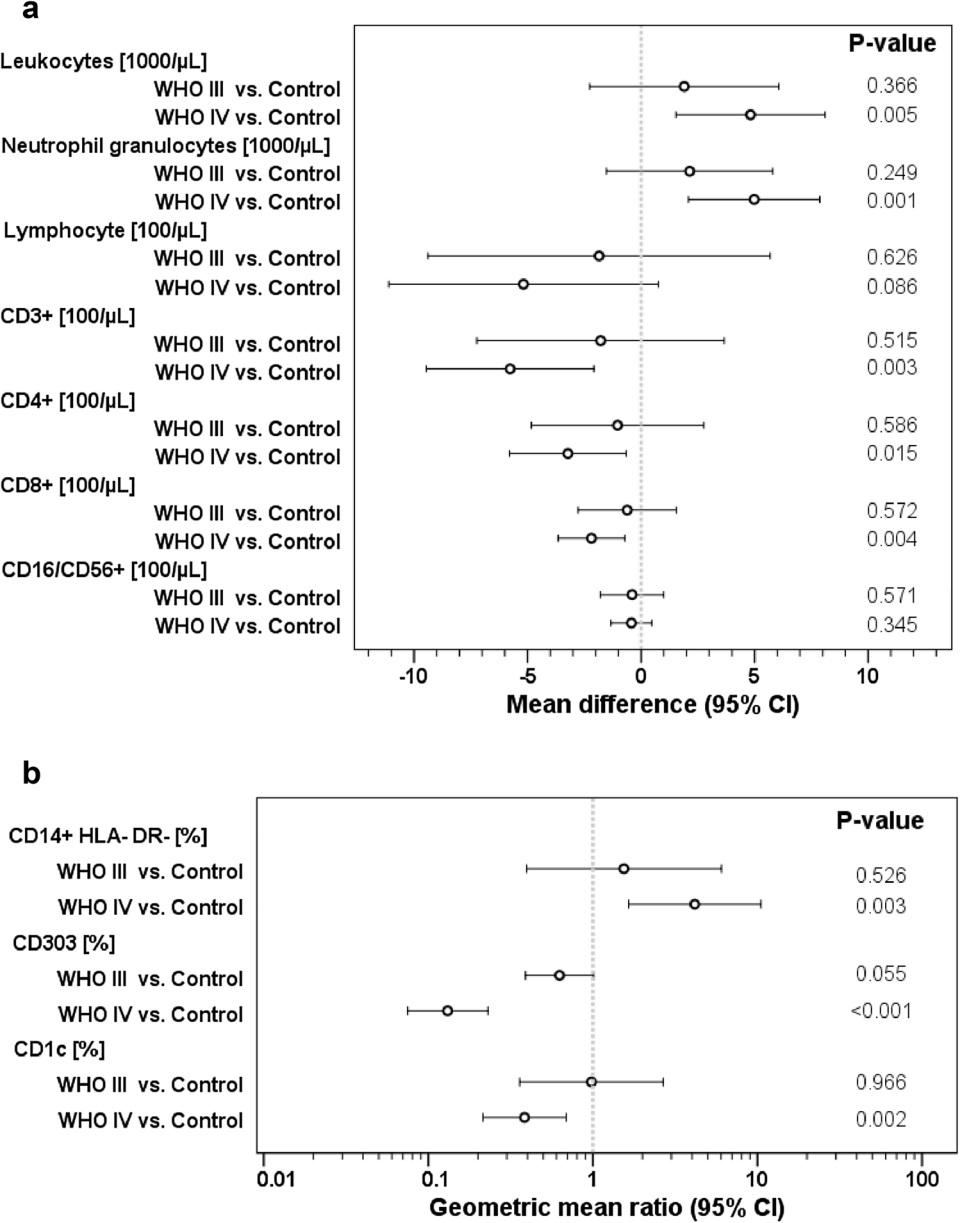
Leukocytes and different leukocyte subsets in peripheral blood of HGG patients (*n* = 61) and healthy controls (*n* = 9). **a** Leukocytes, neutrophils and lymphocytes were measured using an automated hematology counter or by flow cytometry. **b** DC and lymphocyte subsets were analyzed by standard flow cytometry. Inter-group differences were evaluated by one-way ANOVA

Leukocyte and neutrophil numbers were significantly increased in HGG patients; however, significance was only reached for WHO IV° patients (Fig. 1a). Since more WHO IV° patients received corticosteroids, we performed an univariate ANOVA including the parameters WHO grade and dexamethasone, which showed a significantly higher impact of dexamethasone (effect estimates for leukocytes: dexamethasone + 4189/µl (95% CI 1383–6995), *p* = 0.004, and WHO IV° + 3094/µl (95% CI − 360 to 6548), *p* = 0.078; effect estimates for neutrophils: dexamethasone + 4191/µl (95% CI 1660–6724), *p* = 0.002, and WHO IV° + 3349/µl (95% CI 232–6467), *p* = 0.036).

In contrast to neutrophils, total lymphocytes as well as T-cell counts were decreased in HGG patients (Fig. 1a), depending on histology (significant only in WHO IV°). Univariate ANOVA revealed no correlation between dexamethasone but between WHO IV° histology and CD3^+^ and CD8^+^ cell numbers (effect estimates for CD3^+^: for WHO IV° − 623/µl (95% CI − 962 to − 284), *p* = 0.001, and for dexamethasone − 55/µl (95% CI − 338 to 228), *p* = 0.695; effect estimates for CD8^+^: for GBM − 227/µl (95% CI − 397 to − 56), *p* = 0.011, and for dexamethasone − 26/µl (95% CI − 168 to 116), *p* = 0.716). As a consequence of the above mentioned data, the neutrophil/lymphocyte ratio was increased in WHO IV° patients (not shown).

Neither T_reg_ cells (defined as CD3^+^CD4^+^25^+^CD127^neg^) nor any other T-cell subset (naive, central memory, effector memory, and T_EMRA_) showed changes in HGG patients compared to healthy controls (not shown).

In order to exclude that the heterogeneity of our cohort I impacted the results, we grouped patients with primary *vs*. relapsed disease and those with a putatively better prognosis (WHO III° and children) against adult WHO IV° cases. The interaction *p*-value showed no statistically significant interaction between these parameters in our cohort (supplementary Table 1). For CD3^+^ and CD4^+^ T cells we found a trend for interaction (*p* < 0.2), and a subsequent subgroup analysis indicated that T-lymphopenia might be more profound in WHO IV° adults than in WHO III° patients or children (supplementary Table 1). This trend remains to be confirmed in larger cohorts powered for such comparison.

### Monocytic and myeloid-derived suppressor cells in GBM tissue and peripheral blood

Frequencies of HLA-DR^low/neg^ monocytes in HGG patients were increased, whereas the pDC and CD1c^+^ conventional dendritic cells type 2 (cDC2) subsets were significantly reduced in HGG patients (Fig. 1b). For the other analyzed MDSC phenotypes, no difference to healthy controls neither pre- nor post-operatively could be detected. Use of dexamethasone had no influence on HLA-DR^low/neg^ monocytes or pDC/cDC2 in univariate ANOVA.

**Fig. 2:**
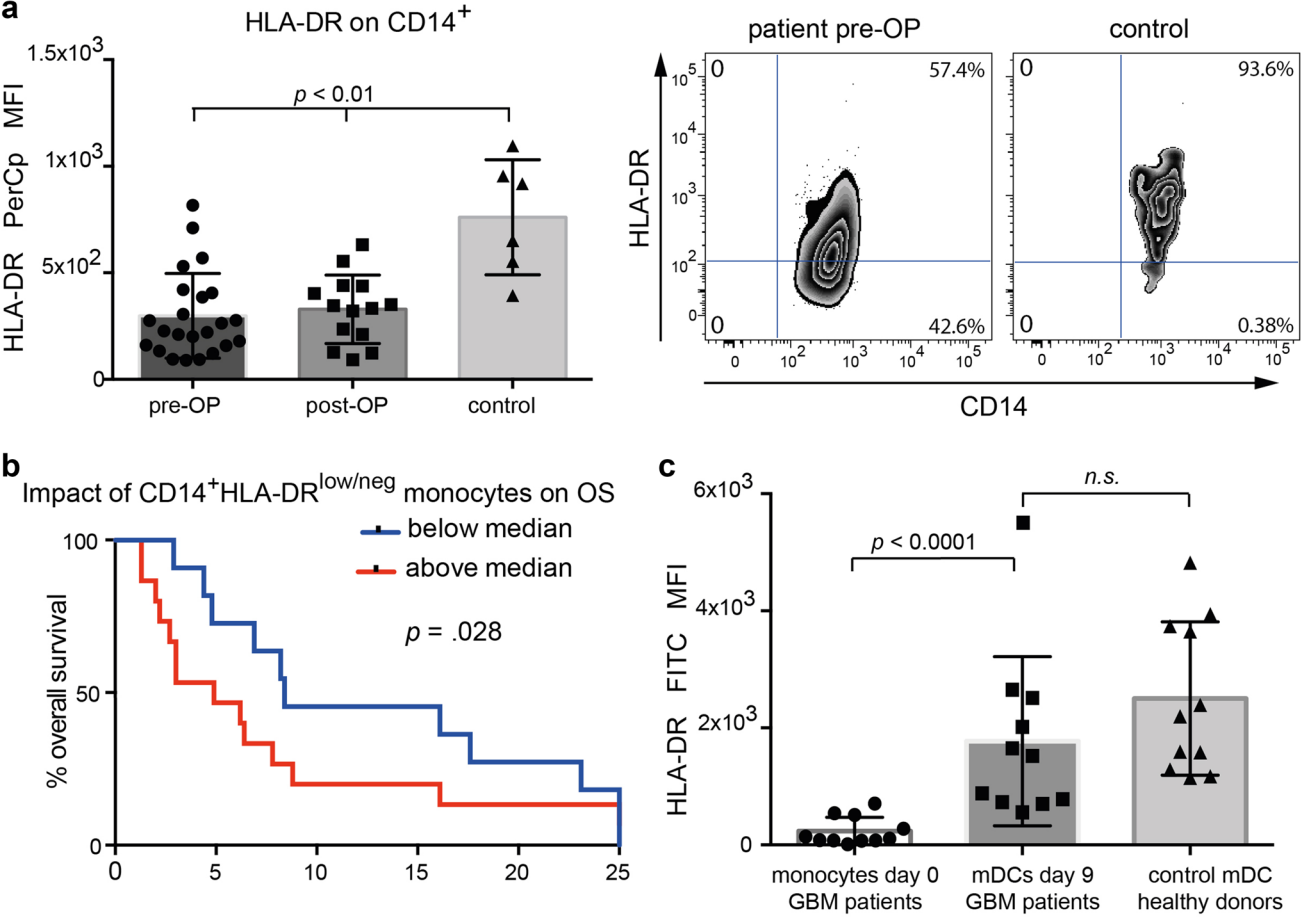
HLA-DR expression on monocytes of HGG patients. **a** HLA-DR on SSC^intermed^CD14^+^ monocytes before and after glioma resection in comparison to healthy controls. Right panels show representative dot plots. **b** Kaplan–Meier plot of overall survival in subgroups defined by median split of the frequency of MDSCs in peripheral blood. **c** HLA-DR on monocytes from HGG patients prepared in vitro for vaccination purposes, in comparison to matured DCs from healthy individuals. Monocytes were analyzed at baseline (after apheresis) and after 9 days maturation with IL-4, GM-CSF, TNFα, and IL-1β

Glioma-associated modulation of HLA-DR expression could also be confirmed by a significantly lower MFI for HLA-DR on patient’s monocytes compared to controls (Fig. 2a, left column). Surgical resection of the tumor mass had no immediate effect on HLA-DR expression levels, as an early postoperative control after a median of 6 days showed no improvement (Fig. 2a, middle column). Correlation of clinical outcome data with MDSC levels revealed that this cell population indeed had a detrimental effect on outcome as patients with HLA-DR^low/neg^ frequencies above the median had a 3.1-fold faster progression (95% CI 1.4–6.8, *p* < 0.006) and died 2.4-fold faster (95% CI 1.1–5.3, *p* = 0.028) than patients with lower HLA-DR^low/neg^ frequencies (Fig. 2b). This data remained constant after adjusting for risk factors: 2.5-fold faster progression, (95% CI 1.1–5.7, *p* = 0.024), and 1.9-fold faster death (95% CI 0.9–4.3, *p* = 0.113). Finally, we wanted to investigate whether suppressive molecules such as Arginase II, CD39 or CD33 play a role in the microenvironment of HGGs. To this end, we analyzed an independent cohort of *n* = 22 confirmed primary glioblastoma samples from adult patients obtained from our neuropathological tumor bank. In contrast to CD68^+^, CD39 was found only on tumor vasculature, whereas Arginase II and CD33 expression was virtually absent (supplementary Fig. 2).

Monocytes from the 11 HGG patients in our cohort II showed a significant upregulation of HLA-DR upon maturation with TNFα/IL-1β (Fig. 2c), which was not different from that of DCs generated from healthy donor’s monocytes, demonstrating that under GMP-manufacturing conditions monocytes from HGG-patients are suitable DC-precursors.

### Functionality of peripheral plasmacytoid DC subpopulations in GBM patients

**Fig. 3:**
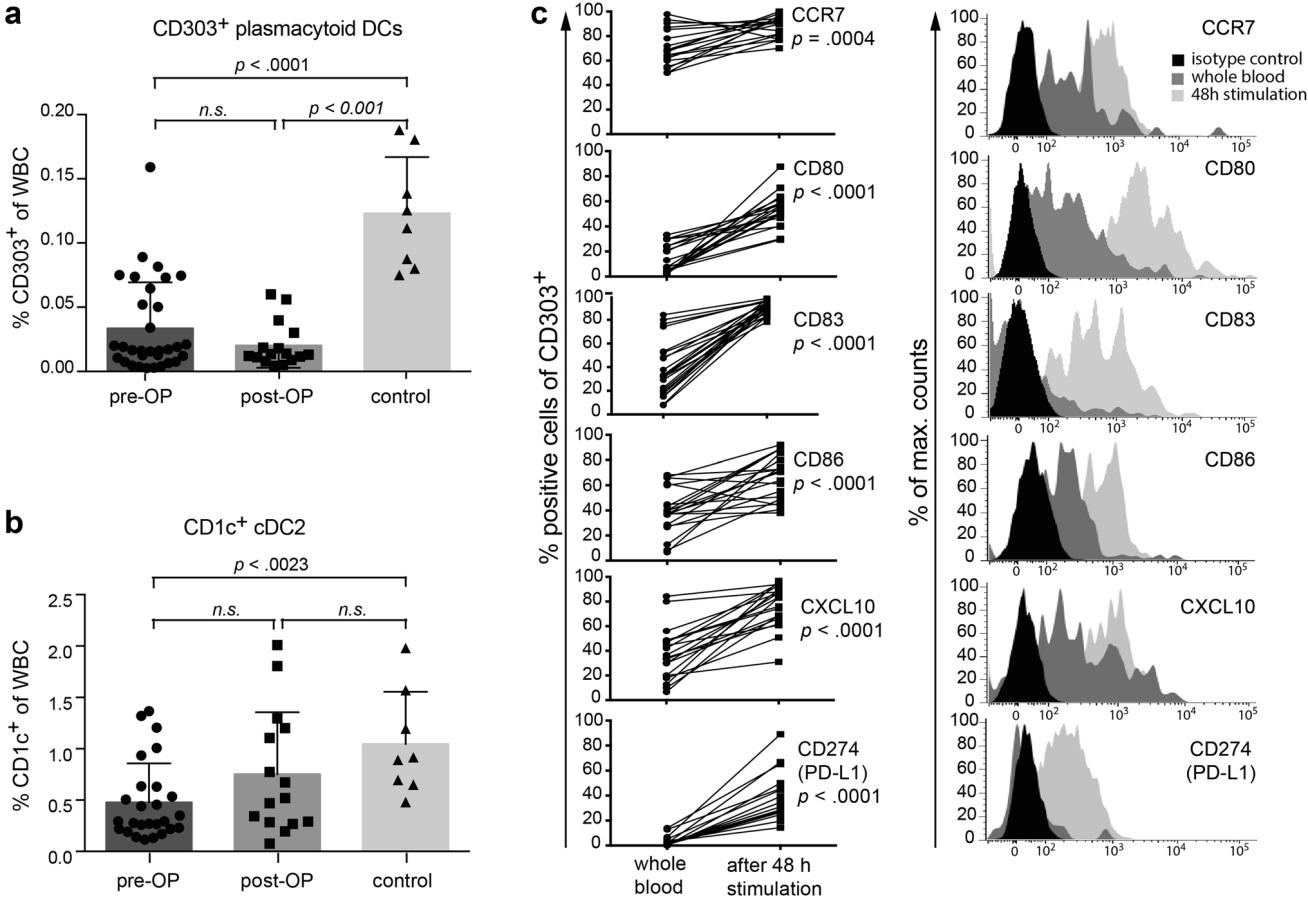
Phenotype of plasmacytoid and cDC2 in peripheral blood of HGG patients before (*n* = 31) and after neurosurgery (*n* = 15) compared to healthy controls (*n* = 9). **a** pDCs were identified as SSC^low^/CD303^+^, **b** cDC2 as SSC^low^/CD1c^+^. **c** pDCs were enriched via BDCA-4 microbeads and stimulated in the presence of imiquimod and IL-3. After 48 h, cells were harvested and analyzed for upregulation of maturation markers by flow cytometry

As mentioned above, pDCs were virtually absent in the majority of HGG patients (Figs. 1b, 3a). Tumor removal had no immediate impact on the number of pDCs (Fig. 1a, middle column). Similar observations were made for cDC2 (Figs. 1b, 3b); however, cDC2 increased postoperatively to some extent so that the difference to healthy controls was not significant anymore (Fig. 3b). Expression of various activation markers on HGG-cDC2 was not different from healthy controls (supplementary Fig. 3, lower graphs). In contrast, pDCs from HGG patients tended to express higher marker levels than pDCs from healthy individuals. This reached statistical significance for CD80 before and after surgery, for IP-10 before surgery and for CD83 and PD-L1 after surgery (supplementary Fig. 3, upper graphs).

In order to examine whether the remaining pDCs in HGG patients are functional, we stimulated them for 48 h in vitro with IL-3 and imiquimod. Costimulatory molecules such as CD80 and CD86, as well as chemokine receptors (CCR7, CXCL10), and inducible markers (CD83, PD-L1) were unequivocally and significantly upregulated (Fig. 3c), indicating that remaining pDCs in HGG patients are functionally intact.

### Biomarker assessment in HGG patients

**Fig. 4:**
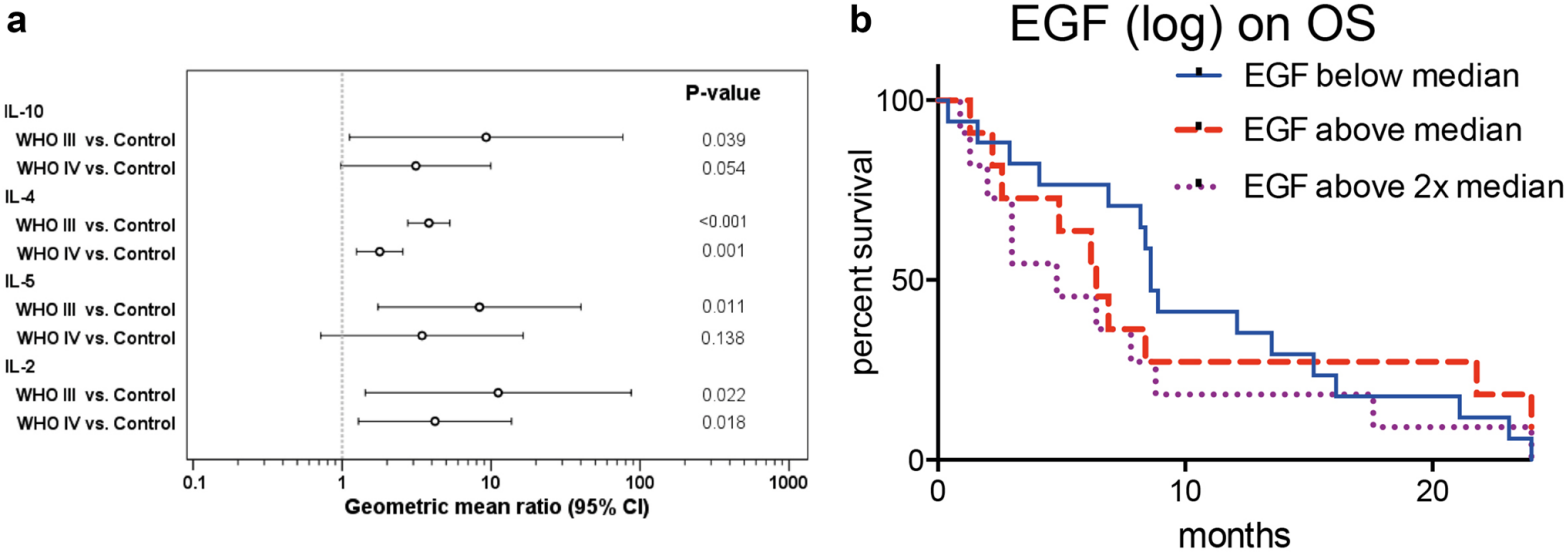
Serum biomarkers in peripheral blood of HGG patients compared to healthy controls. **a** Biomarkers with a significant difference between HGG patients and healthy controls. Inter-group differences were evaluated by one-way ANOVA. **b** Kaplan–Meier plot of overall survival in subgroups defined by EGF serum levels split into tertiles

Of the 34 analyzed potential biomarkers, only 4 (IL-2, IL-4, IL-5, IL-10) proved to be elevated in HGG patients (Fig. 4a). IL-4 and IL-5 were significantly lower in WHO IV° than in WHO III° patients (2.14-fold and 2.43-fold lower, *p* < 0.001 for IL-4 and IL-5, respectively). Correlating biomarker levels with clinical outcome parameters we found that none of the above markers showed an association with survival. Instead, a significant impact of EGF on OS could be described: a doubling of EGF serum levels was associated with a 1.3-fold increased risk for death (95% CI 1.0–1.6, *p* = 0.021, Fig. 4b). This data remained constant after adjusting the hazard ratio for clinical risk factors: 1.23-fold incrased risk for death (95% CI 1.00–1.52, *p* = 0.049) for a doubling in EGF serum levels.

### Clinical characteristics of vaccinated HGG patients and adverse events

**Fig. 5:**
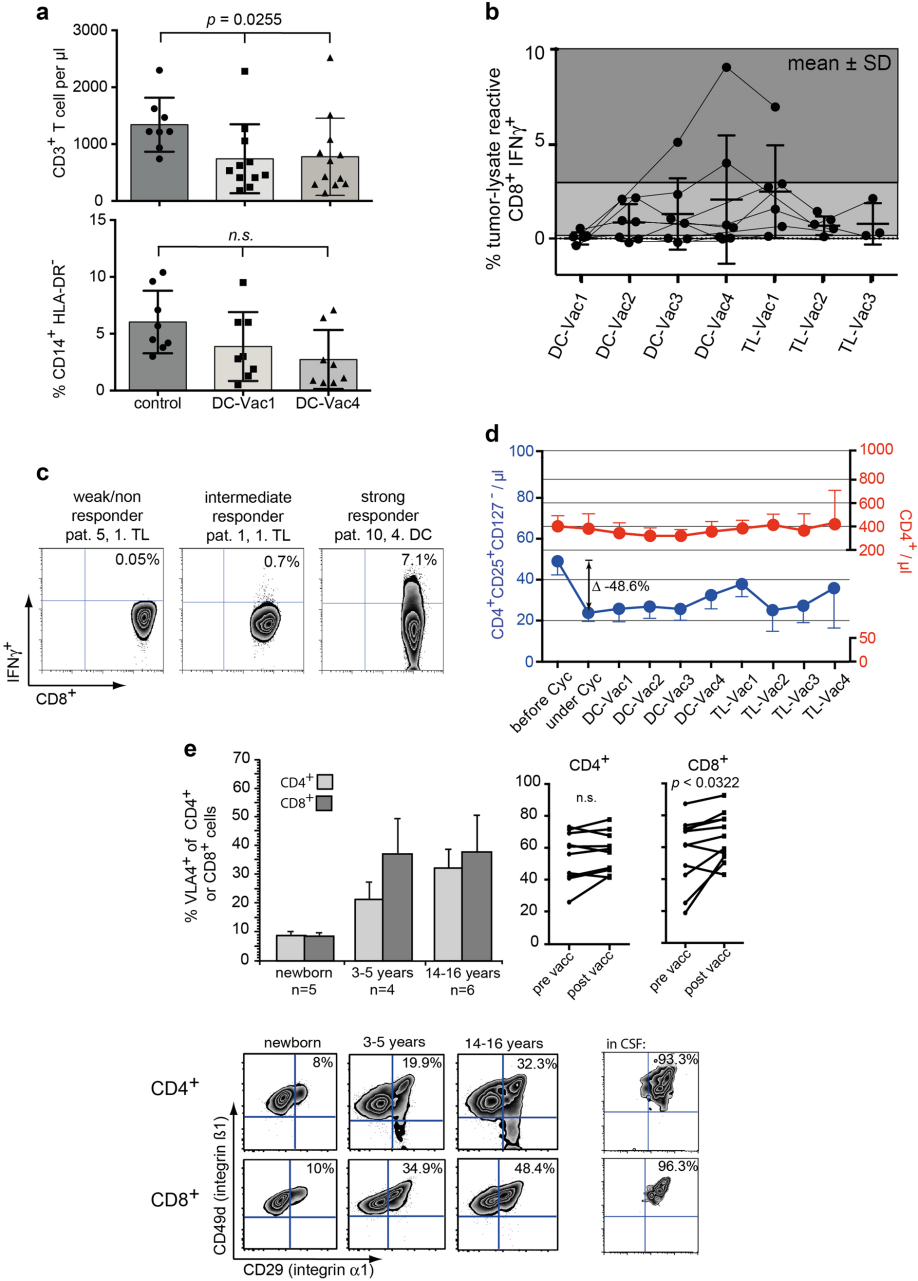
Clinical and immunological data of 11 relapsed HGG patients receiving T_reg_-depletion followed by a therapeutic vaccine. **a** T-lymphopenia (upper graph) and frequency of monocytic MDSCs (lower panel) at the beginning and after 4 vaccines compared to healthy controls. **b** Summary of IFNγ^+^ tumor-specific T cells before (DC-Vac1) and during vaccination. The gray scale corresponds to areas of weak, intermediate and strong responders. **c** Representative examples of weak, intermediate and strong responders. Cells were gated on SSC^low^CD3^+^CD8^+^. **d** Time course of absolute CD4^+^ T cells (in red) and CD4^+^CD127^−^CD25^+^ T_reg_ cells (in blue) during and after metronomic cyclophosphamide. Note different scaling of the right and left *y* axis. Data are shown as mean values ± SEM. **e** VLA-4 expression on CD4^+^ and CD8^+^ T cells during childhood and adolescence. Column bars show mean ± SEM values; zebra plots in the lower panels are representative examples of VLA-4 expression on CD4^+^ and CD8^+^ T cells of the respective age group as well as in CSF. The upper right line graphs display development of VLA-expression on CD4^+^ and CD8^+^ T cells before (DC-Vac1) and after (TL-Vac1) vaccination

The second cohort of HGG patients consisted of 11 patients with relapsed tumors who were eligible for second resection (Table 1, bottom). These patients received immunotherapy (vaccination with autologous, tumor-lysate pulsed DCs after depletion of T_reg_ with metronomic cyclophosphamide) as the sole form of relapse treatment until further progression. The four weekly vaccinations, followed by monthly boost vaccines with tumor lysate, were well tolerated. All patients experienced mild and transient swelling and itching at the local injection site. Two patients reported headache or deterioration of their neurological symptoms 6–8 days after the vaccine. In both cases symptoms were transient and responded well to corticosteroids. Interestingly, neuroimaging revealed that occurrence of these two adverse events was associated with further local progression and significant edema at the tumor site. The size of the vaccination cohort was not powered to demonstrate efficacy; however, a 6-month OS in the immunotherapy group of 100% and an OS of 14.9 ± 13.4 months are encouraging (Table 1). Importantly, under immunotherapy, increased frequencies of HLA-DR^neg^ monocytes (Fig. 5a, lower graph) as well as reduced pDCs and cDC2s (not shown) were no longer detectable, whereas T cells were still lower than in the healthy control group (Fig. 5a, upper graph).

### T_reg_ under metronomic cyclophosphamide

Metronomic cyclophosphamide was generally well tolerated. In two patients, a dose reduction was necessary due to a drop of leukocytes < 2000/µl. Under this metronomic schedule, absolute numbers of T_reg_ could be reduced by 48% (Fig. 5d), whereas total CD4 counts remained constant. Approx. 4 weeks after cessation of cyclophosphamide, a rebound of T_reg_ could be observed (Fig. 5d).

### Immune responses and T-cell subsets under vaccination

Despite persisting T-lymphopenia also in vaccinated patients (Fig. 5a, upper graph), measurable IFNγ-responses were detected in 9 out of 10 analyzed patients, which seemed to peak between the 2nd DC- and 1st TL-vaccine (Fig. 5b). 2/10 patients showed no or only a minor response (≤ 0.1% CD3^+^CD8^+^IFNγ^+^), 6 patients displayed intermediate responses (0.2–3%), and 2 patients had high-frequency responses (3–9%, Fig. 5c). A possible cytotoxic effect of these tumor-lysate reactive T-cells on glioma cells presumes that T-cells are endowed with key entry molecules into the CNS. Very late antigen 4 (VLA-4), a α_4_β_1_ integrin dimer (CD49d/CD29) had been described as one of the prime factors for CNS homing [33], and in fact, normal T-cells in CSF are all VLA-4^+^ (Fig. 5e). Looking at VLA-4 expression on CD4^+^ and CD8^+^ T-cells in different age groups we found that VLA-4 is dynamically upregulated during childhood and adolescence (Fig. 5e), most likely due to increasing contact with environmental antigens and formation of memory T-cell responses. Next, we analyzed whether vaccination with glioma-lysate pulsed DCs had any impact on VLA-4 expression of peripheral T cells. Indeed, we found a significant increase of VLA-4 expression on CD8^+^ but not on CD4^+^ T-cells during the vaccination course (Fig. 5e).

## Discussion

Glioma cells use many different pathways to circumvent detection and eradication by the immune system as recently reviewed by Nduom et al. [34]. Systemic immunosuppression may hinder the development of anti-tumor immune responses, as priming of glioma-specific cytotoxic T-cells has to occur outside the CNS in secondary lymphatic organs. In fact, none of the 11 patients in our immunotherapy group showed a substantial immune response against tumor lysate before initiation of the vaccination schedule, underlining that endogenous immune responses against solid tumors are rare events [35]. Our data confirmed previous findings in glioma patients: a profound T-lymphopenia and a general deficit in lymphocyte function [3, 36–38], the increased presence of monocytic MDSCs [5], dysregulation of serum cytokines [39], as well as an elevated neutrophil-to-lymphocyte ratio [40]. Only the increase in neutrophils was connected to the use of corticosteroids; all other changes in cellular subsets were connected to HGG histology (WHO III° vs. IV°), suggesting a link between immunosuppression and glioma biology. Noteworthy, glioma-associated lymphopenia was already detectable at primary diagnosis with a trend for lower lymphocyte values in adult patients with primary WHO IV° tumors, indicating that subsequent radiochemotherapy cannot be the sole reason for this phenomenon.

Our data underline the clinical importance of the myeloid/monocyte compartment in HGGs: higher numbers of monocytic MDSCs and EGF serum levels above the median were negatively associated with overall and for MDSCs also with progression-free survival. As monocytes are a prime source of EGF [41], both clinical risk factors can be attributed to the MDSC pathway. A direct glioma–MDSC interaction has been postulated experimentally as glioma cell supernatant as well as a direct cell–cell contact between glioma cells and monocytes induce a MDSC phenotype [7], which is also functionally suppressive by inhibiting T-cell proliferation [5, 42]. However, conflicting data exist about the presence of MDSCs in the glioma microenvironment: Prosniak et al. have described that MDSC infiltration is present in all samples and increases with tumor grade [43], whereas Gustafson et al. could find MDSCs only in 48% of GBM samples [5]. In an independent set of GBMs from our tumor bank, we were unable to detect expression of suppressor molecules such as Arginase II or CD39 in glioma tissue. In contrast, the isoform Arginase I has been repeatedly detected in glioma-infiltrating myeloid cells [44]. Therefore, for a comprehensive and differentiated view on MDSCs, macrophages and other infiltrating innate cells in the glioblastoma microenvironment a broad panel of markers has to be analyzed.

Depletion of pDC/cDC2 represents a well-known immune escape mechanism in several cancer entities such as melanoma and breast cancer. Here, we describe this phenomenon also in HGGs. Both types of dendritic cells play unique roles in immunity and especially cDC2 are capable of efficient cross-priming of CTL responses from naive CD8^+^ T cells [45]. In contrast to monocytes, pDC and cDC2 were only decreased in numbers. They expressed high levels of costimulatory molecules and pDCs adequately upregulated activation markers upon in vitro stimulation. Regarding the altered cytokine levels observed in our patients, a very similar cytokine pattern with elevated levels of IL-2, IL-4, and IL-13 was found in serum of HGG patients with high frequencies of MDSCs [46]. Elevated IL-2 levels could be derived from activated peripheral blood T-cells, as the described T-cell exhaustion with deficient IL-2 production seems to be restricted to tumor infiltrating T-cells [47]. However, since neither DC subsets nor cytokine levels showed an association with OS or PFS, the clinical relevance of these two latter findings has to be questioned.

In view of the multilayered complexity of immunosuppression in HGGs, an intervention to interrupt this network seems highly desirable. Although some groups have reported about MDSC reduction by inhibition of COX-2 [11] or all-trans retinoic acid [29], far more data exist on the synergistic effect of T_reg_ depletion and therapeutic cancer vaccines [48, 49]. Cyclophosphamide has been known for almost 30 years to mediate immunity-driven cancer regression [22] and in a metronomic schedule it reduces the frequency of T_reg_ [26]. Beyond numerical depletion, cyclophosphamide also induces apoptosis and profound functional inhibition in remaining T_reg_ [50]. Furthermore, it can restore perturbed DC homeostasis [51] and positively influence the host microenvironment [13], resulting in infiltration with non-suppressive myeloid cells [52]. In renal cell carcinoma patients, a single dose of cyclophosphamide prevaccine reduced T_reg_ by 20%, inhibited T_reg_-proliferation, and resulted in prolonged survival in those patients displaying a positive immune response [24]. In our HGG cohort, metronomic cyclophosphamide was well tolerated and not associated with myelosuppression. We observed *a* > 40% reduction in T_reg_ numbers in the peripheral blood, which, after withdrawl, lasted for several weeks followed by a rebound. At the time of vaccination and throughout follow-up, frequencies of monocytic MDSCs and pDC/cDC2 had normalized and were not different from those of healthy controls anymore. Most importantly, using this regimen we were able to detect measurable IFNγ-T-cell responses in 90% of analyzed patients, which is higher than the reported average of 50.3% (33–66%) immune responders in other HGG-vaccination trials [14–21]. IFNγ-responses seemed to diminish under lysate-boosts, which could indicate that lysate given without adjuvant DCs is not able to maintain induced immune responses or that tolerizing events appeared. The relatively young age of our immunotherapy cohort also might have contributed to the high frequency of immune responses, as the latter might correlate with residual thymic function [53]. Interestingly, the two strong responders (3–9% IFNγ^+^ T cells) presumably had a high antigen load in situ (pat. 3 proved to have an early progression under vaccination, pat. 10 a substantial residual tumor after incomplete resection), and both experienced neurological side effects 6–8 days after the vaccine (headache in 2/2, deterioration of hemiparesis in 1/2, which responded rapidly to corticosteroids). The pilot phase of our *HIT-HGG Rez Immunovac* trial was not powered for efficacy analysis; however, a 6-month OS of 100% is encouraging, considering that these relapsed GBM patients received only surgical resection and immunotherapy. Clinical data clearly await confirmation in the running trial.

A prerequisite for the proposed mechanism of action of an antiglioma vaccine is the induction of T-cells endowed with CNS-homing receptors such as α_4_β_1_-integrin (VLA-4). Experimental evidence from murine models has shown that VLA-4 is upregulated on T cells upon antigen-specific activation by DCs [54], but the site of DC injection and regional lymph node localization seem to play important roles in mice as well [55]. The age-dependent augmentation in VLA-4 expression on pediatric T cells confirms that also in humans an increasing antigen-experience is paralleled by upregulation of this integrin. Importantly, we can demonstrate that the intradermal application of tumor-lysate loaded DCs in the upper arm of glioma patients results in a significant upregulation of VLA-4 on CD8^+^ T cells. It is an interesting anecdotal observation that IL-4, which was elevated in all HGG patients, inhibits VLA-4 expression on CD4^+^ T cells [56]. Therefore, effects of CD4^+^ T cells might be restricted to T-cell help in lymphoid tissues in our patients. In summary, these data strengthen the suitability of our approach to de novo induce tumor-specific T-cells with CNS homing properties.

One limitation of our study is that we could not precisely determine the time point when the normalization of MDSCs and pDC/cDC2 subsets occurred. Tumor surgery alone had no immediate effect on these parameters as one early follow-up timepoint in cohort I 1 week after surgery revealed. Only three patients from cohort I directly proceeded to immunotherapy. All other patients have been referred to our center for DC vaccination and we could not get samples from the time before metronomic cyclophosphamide started. Therefore, a closer look at the timely association between immunosuppressive features and diagnosis, surgery, initiation of T_reg_-depletion and vaccination is one of the tasks for *HIT-HGG Rez Immunovac* trial.

In conclusion, our comprehensive analysis of leukocyte subsets and serum biomarkers in peripheral blood of HGG patients revealed several distinct mechanisms of systemic immunosuppression, underlining the clinical relevance of monocytic MDSCs in HGGs. In relapsed HGG patients, a DC-based vaccine preceded by one course of metronomic cyclophosphamide not only reduced T_regs_ but also led to a normalization of monocytic MDSC as well as pDC/cDC2 subsets and allowed induction of tumor-specific CD8^+^VLA-4^+^ T cells in > 90% of patients. Our approach may, therefore, represent a first step to reverse glioma-associated immunosuppression, thereby enhancing efficacy of therapeutic vaccines.

## Electronic supplementary material

Below is the link to the electronic supplementary material.

Supplementary material 1 (PDF 7366 KB)

## Abbreviations

7-AAD: 7-aminoactinomycin
BDCA-4: Blood dendritic cell antigen 4, neuropilin
CCR: Chemokine receptor
cDC2s: Conventional dendritic cells type 2
CI: Confidence interval
CNS: Central nervous system
CXCL: Chemokine ligand
EGF: Epidermal growth factor
Fas: First apoptosis signal receptor
FasL: First apoptosis signal ligand
FGF: Fibroblast growth factor
GBM: Glioblastoma multiforme
HGF: Hepatocyte growth factor
HGG: High-grade glioma
IL-1RA: Interleukin 1 receptor antagonist
IP-10: Interferon gamma-induced protein 10, CXCL10
pDCs: Plasmacytoid dendritic cells
RANTES: Regulated on activation, normal T cell expressed and secreted, CCL5
SSC: Sideward scatter
T_EMRA_: Effector memory T cells with CD45RA expression
TL: Tumor lysate
T_reg_: Regulatory T cells
VLA-4: Very late antigen 4
