# Supplementary material for: High-grade glioma associated immunosuppression does not prevent immune responses induced by therapeutic vaccines in combination with Treg depletion
Source: Cancer Immunol Immunother. 2018 Jul 27;67(10):1545–58. doi: 10.1007/s00262-018-2214-0 (PMC6182405; doi:10.1007/s00262-018-2214-0)
Supplement: Supplementary file 1 — Supplementary material 1 (PDF 7366 KB) [file 262_2018_2214_MOESM1_ESM.pdf]

supplementary Figure 1

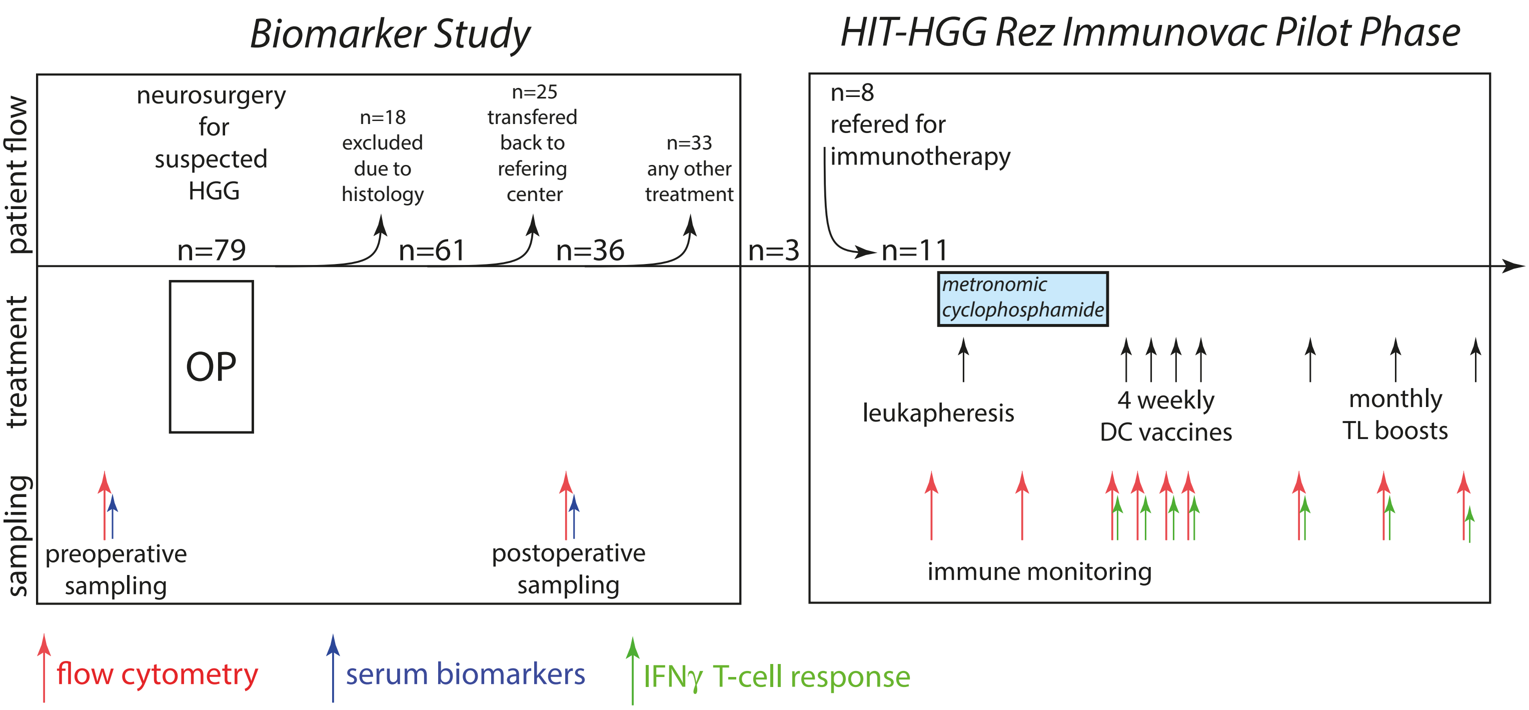

supplementary Figure 1.
Patient flow as well as diagnostic and therapeutic interventions in the biomarker study (cohort I) and in the pilot phase of the *HIT-HGG Rez Immunovac* trial (cohort II).

supplementary Figure 2

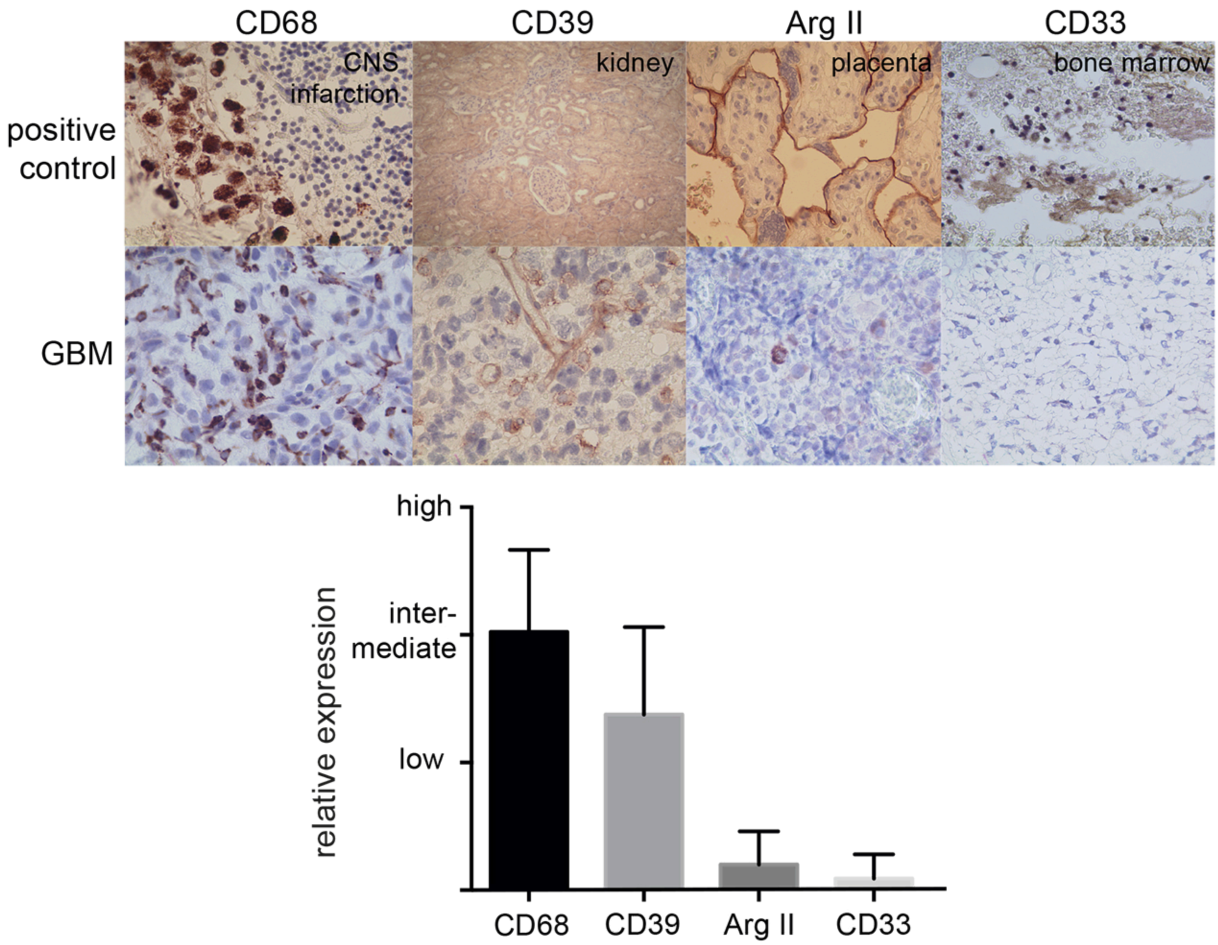

supplementary Figure 2:
Expression of markers associated with MDSC function (CD39, Arginase II) in GBM tissues. N=21 confirmed and anonymized tissues from adult patients with a primary GBM were obtained from a neuropathological tumor bank.CD68 identified microglia, CD33 myeloid/monocytic cells in general. Tissues from kidney, placenta, CNS infarction, and bone marrow served as a positive staining control.
Paraffin-embedded samples (thickness 2 µm) of glioblastoma tissue were stained with standard immunohistochemistry procedures against CD68 (Dako, Glostrup, Denmark; dilution 1:25), CD33 (Thermo Scientific, Rockford, USA; dilution 1:50), CD39 (Atlas-Antibodies, Voltavägen, Sweden; dilution 1:100), Arginase II (Atlas-Antibodies; 0.09 mg/ml, dilution 1:600). One day after primary antibody incubation, slides were stained with AEC-Romulin (Biocare Medical, Concord CA, USA) for 10 min andd counterstained with Mayer`s hemalaun solution.
Expression levels were ranked semiquantitatively by two independent investigators.

supplementary Figure 3

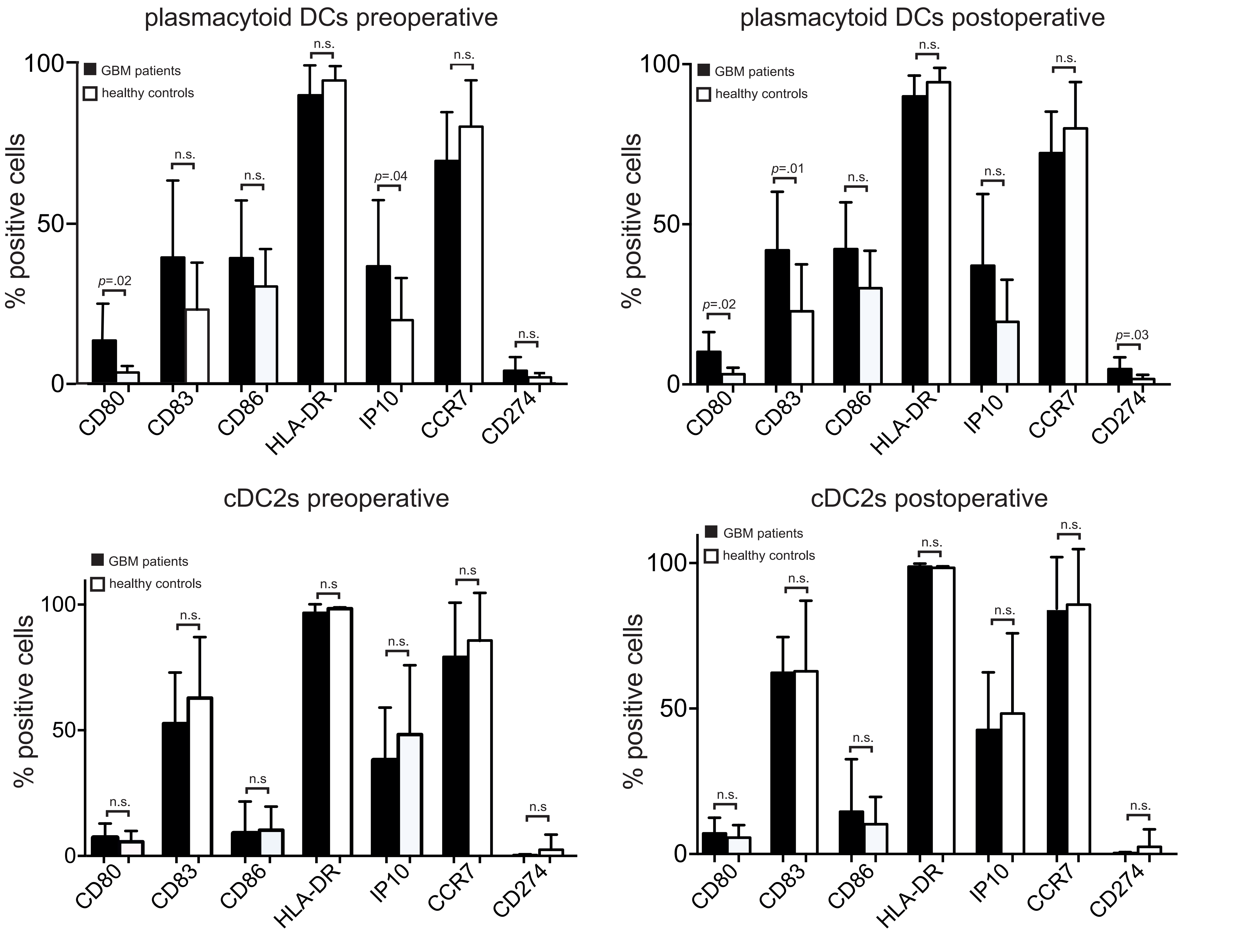

supplementary Figure 3.
Phenotype of plasmacytoid and conventional dendritic cells (cDC2) in peripheral blood of HGG patients before (left graphs, n=22) and after neurosurgery (right graphs, n=11) compared to healthy controls (n=9). In a standard lyse/wash-procedure, white blood cells were stained with the respective monoclonal antibodies and analyzed on a FACS Canto II. Plasmacytoid DCs were identified as $SSC^{low}/CD303^{+}$, myeloid DCs as $SSC^{low}/CD1c^{+}$ events, and the respective surface marker expression was determined on gated cells using FlowJo 9.6.

Supplementary Table 1. Difference in mean biomarker levels in patients with tumor relapse compared to those with primary tumor.

**Biomarker variable**	**Mean difference (95% CI) of relapse vs. primary**	**P-value for relapse** [a]	**Interaction P-value** [b]
Leukocytes [1000/µL]	–6.3 (–8.5 to –4.2)	<0.001	0.825
Neutrophil granulocytes [1000/µL]	–5.7 (–7.6 to –3.7)	<0.001	0.805
Lymphocytes [100/µL]	–2.0 (–6.5 to +2.6)	0.387	0.405
CD3+ [100/µL]			
All patients	–0.2 (–3.4 to +3.1)	0.914	
WHO IV° adults	–4.0 (–7.9 to +0.1)	0.043	0.112
WHO III° adults + IV° children	+1.1 (–3.9 to +6.2)	0.654	
CD4+ [100/µL]			
All patients	–0.6 (–2.6 to +1.5)	0.573	
WHO IV° adults	–3.0 (–5.5 to –0.5)	0.019	0.051
WHO III° adults + IV° children	+1.1 (–2.2 to +4.4)	0.507	
CD8+ [100/µL]	+0.1 (–1.3 to + 1.5)	0.870	0.566
CD16+/CD56+ [100/µL]	–0.7 (–1.5 to +0.1)	0.069	0.730
	**Geometric mean ratio (95% CI)**		
CD14+ HLA– DR– [%]	0.9 (0.3 to 2.4)	0.800	0.953
CD303 [%]	3.3 (1.4 to 7.7)	0.009	0.325
CD1c [%]	0.6 (0.3 to 1.4)	0.226	0.853
IL-10	1.5 (0.7 to 3.6)	0.313	0.240
IL-4	1.2 (0.8 to 1.7)	0.466	0.477
IL-5	1.2 (0.7 to 2.1)	0.425	0.556
IL-2	1.3 (0.6 to 2.7)	0.479	0.878

[a] Results in subgroup analysis of WHO IV adults and the pooled WHO III adults and WHO IV children are provided only when the interaction *p*-value was < 0.2.

[b] Test of the null hypothesis that mean differences between relapses and primary tumors were the same in WHO IV adults and the pooled group of WHO III adults and WHO IV children.
